# Dopamine-dependent changes of cortical excitability induced by transcranial static magnetic field stimulation in Parkinson’s disease

**DOI:** 10.1038/s41598-017-04254-y

**Published:** 2017-06-28

**Authors:** M. Dileone, M. C. Carrasco-López, J. C. Segundo-Rodriguez, L. Mordillo-Mateos, N. López-Ariztegui, F. Alonso-Frech, M. J. Catalan-Alonso, J. A. Obeso, A. Oliviero, G. Foffani

**Affiliations:** 1 CINAC, Hospital Universitario HM Puerta del Sur, Universidad CEU San Pablo, Móstoles, Madrid, Spain; 2 Hospital Nacional de Parapléjicos, SESCAM, Toledo, Spain; 3 Servicio de Neurología, Hospital Virgen de la Salud, SESCAM, Toledo, Spain; 4 Hospital Clínico San Carlos, Madrid, Spain

## Abstract

Transcranial static magnetic field stimulation (tSMS) is a recent low-cost non-invasive brain stimulation technique that decreases cortical excitability in healthy subjects. The objective of the present study was to test the ability of tSMS to modulate cortical excitability in patients with Parkinson’s disease. We performed a randomized double-blind sham-controlled cross-over study to assess cortical excitability before and immediately after tSMS (or sham) applied for 10 min to the more affected motor cortex of patients with Parkinson’s disease. Cortical excitability was quantified by the amplitude of motor evoked potentials (MEPs) elicited by single-pulse transcranial magnetic stimulation (TMS). tSMS significantly decreased MEP amplitudes in patients OFF medication (after overnight withdrawal of dopaminergic drugs), but not ON medication (after an acute dose of levodopa). The between-patients variability of tSMS-induced changes was significantly greater ON medication. The variability ON medication could be partly explained by disease progression, i.e. the more advanced the patient, the more likely it was to observe a switch from inhibitory tSMS plasticity OFF medication to paradoxical facilitatory plasticity ON medication. These results suggest that tSMS induces dopamine-dependent changes of cortical excitability in patients with Parkinson’s disease.

## Introduction

Non-invasive brain stimulation (NIBS) techniques offer a set of tools to investigate plasticity phenomena in the human motor cortex^1, 2^. In healthy subjects, cortical plasticity is closely linked to motor learning^3–7^. Accordingly, plasticity induced by both facilitatory and inhibitory NIBS techniques is highly dependent on dopamine^8–11^.

In patients with Parkinson’s disease, a large number of studies reported profound alterations of cortical plasticity induced by excitatory NIBS techniques – with a complex phenomenology that depends on the technique employed, the clinical asymmetry of the patients, the stage of the disease and the administration of levodopa (reviewed in ref. 12) – whereas only few studies investigated cortical plasticity induced by inhibitory NIBS techniques^13–15^. A key observation is that inhibitory cortical plasticity – induced by continuous theta-burst stimulation (cTBS) – can be observed in treated non-dyskinetic patients after overnight withdrawal of dopaminergic medication (OFF) but is disrupted by acute levodopa administration (ON)^15^. Whether the above finding is specific for cTBS or generalizes to other inhibitory NIBS techniques remains unclear.

We recently introduced transcranial static magnetic field stimulation (tSMS), a safe inhibitory NIBS technique that is able to reduce cortical excitability in humans by the simple application of a relatively strong neodymium magnet over the scalp^16–18^. The reduction of cortical excitability induced by tSMS was confirmed by other groups, both in humans^19–22^ and in non-human primates^23^. The aim of the present study was to investigate the ability of tSMS to modulate cortical excitability in patients with Parkinson’s disease.

## Methods

### Patients

We performed a total of 48 experimental sessions in 13 conscious patients with idiopathic Parkinson’s disease (Brain Bank criteria), optimal response to dopaminergic medication (>30% motor improvement as measured by the Unified Parkinson’s Disease Rating Scale part III, UPDRS III), no other main neuropsychiatric co-morbidity, and no MRI-incompatible metal devices in the body. Patients were enrolled at the Movement Disorders Unit of Hospital Virgen de la Salud (Toledo, Spain) and at CINAC, Hospital Universitario HM Puerta del Sur (Móstoles, Spain). All patients gave written informed consent and all procedures were conducted in accordance with the Declaration of Helsinki and approved by the local Ethics Committees (“Comité Ético de Investigación Clínica del Complejo Hospitalario de Toledo” and “Comité Ético de Investigación Clínica del Grupo HM”). Tremor was sufficiently mild not to interfere with the experiments. Overt dyskinesias were not observed during the sessions.

### Experimental design

**Figure 1:**
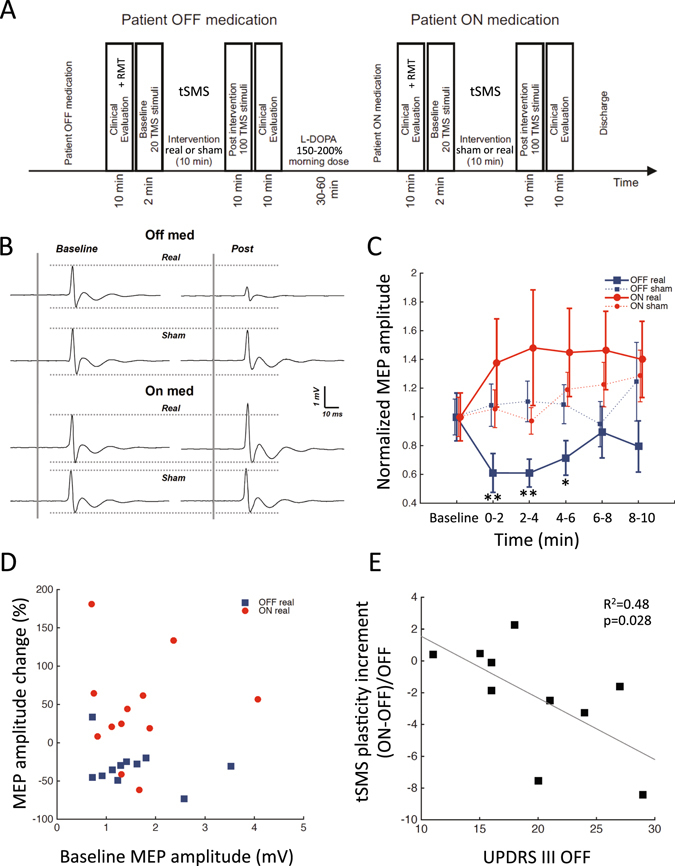
Effects of tSMS on cortical excitability in Parkinson’s disease. (**A**) Experimental protocol. Patients repeated the protocol twice, at least one week apart, exchanging the tSMS intervention (sham vs real). (**B**) Average MEP traces for a single patient, before (baseline) and immediately after (post) real and sham tSMS, OFF and ON medication. (**C**) Average cortical excitability changes, as measured by MEP amplitude (normalized to group-averaged baseline values for visualization purposes), induced by real and sham tSMS in patients OFF medication (blue) and ON medication (red). Error bars are SEM. **p < 0.01, *p < 0.05 (Dunnett) (**D**) Scatter plot of excitability changes induced by real tSMS (100*[0–6 min]/baseline-1, y-axis) vs baseline (x-axis) in patients OFF (blue) and ON (red) medication. (**E**) Scatter plot of relative increment of tSMS plasticity after levodopa (y-axis) vs UPDRS III OFF medication immediately before levodopa intake (x-axis). The best fitting straight line is plotted in gray. tSMS significantly decreased cortical excitability in patients OFF medication, but not ON medication. The between-patients variability of tSMS-induced changes was significantly greater ON compared to OFF medication. The variability ON medication could be partly explained by disease progression, i.e. the more advanced the patient, the more likely it was to observe a switch from inhibitory tSMS plasticity OFF medication to paradoxical facilitatory plasticity ON medication.

We performed a randomized double-blind sham-controlled study with a cross-over design (Fig. 1A). We recorded motor-evoked potentials (MEPs) elicited by single-pulse transcranial magnetic stimulation (TMS) of the motor cortex contralateral to the most affected side, before and immediately after unilateral 10-min tSMS or sham applied for 10 min, both after overnight withdrawal of dopaminergic drugs (OFF) and after an acute dose of levodopa (ON). Each patient underwent 4 experimental sessions: tSMS-OFF, tSMS-ON, sham-OFF, sham-ON. These 4 sessions were performed in two separate days, at least one week apart. In each day the patients underwent one experimental session OFF medication (either tSMS-OFF or sham-OFF, randomized across patients), received the acute dose of levodopa (150–200% of effective morning dose; 200–250 mg), and underwent another experimental session ON medication (either sham-ON or tSMS-ON; if the OFF session was tSMS, the ON session was sham, and viceversa). Patients were also clinically evaluated (UPDRS III) 5 min before and 10 min after each session (tSMS or sham) to assess the switch from the OFF-state to ON-state. We enrolled 15 patients: 11 completed all 4 sessions (post-real OFF data could not be used in one patient), one was studied only in OFF both days, one withdrew after the first day, and two withdrew before the first session. The final sample sizes are as follows: OFF real n = 11; OFF sham n = 13; ON real n = 12; ON sham n = 11. The clinical characteristics of all 13 patients analyzed are included in Table 1.

**Table 1:** Clinical characteristics of the patients studied.

Subject	Gender	Age (years)	Duration of Disease (years)	Duration of Treatment (years)	UPDRS III OFF	UPDRS III ON	Total Levodopa Equivalents (mg/day)
PD1	Male	42	5	4	19.5	10.5	940
PD2	Female	61	5	4	16.5	7.5	853
PD3	Male	64	9	8	27	2	806
PD4	Male	63	7	7	21	2.5	1201
PD5	Female	72	7	7	25.5	9.5	606
PD6	Female	64	6	5	23	7.5	1203
PD7	Male	63	4	3	16	8	1053
PD8	Male	65	7	7	28	13	700
PD9	Male	32	6	6	20.5	9	820
PD10	Female	65	12	12	29.5	7	1025
PD11	Male	67	14	13	38	26	1533
PD12	Male	53	12	10	37	—	1000
PD13	Female	56	6	6	15.5	5.5	670
	**MEAN**	**59.0**	**7.7**	**7.1**	**24.4**	**9.0**	**954.6**
	SD	11.0	3.1	3.0	7.4	6.2	256.9

UPDRS values are the average values per patient across sessions. PD12 was studied only OFF.

Throughout the experiments, patients were seated comfortably in a semi-darkened room, and were instructed to refrain from speaking and to remain awake while in a calm, relaxed state.

### Transcranial static magnetic field stimulation (tSMS)

The magnet used for tSMS was the “Big Magnet” used by Oliviero *et al*.^16^, i.e. a cylindrical neodymium magnet (nickel-plated NdFeB magnet) of 45 mm diameter and 30 mm of thickness, with a weight of 360 g (MAG45r; Neurek SL, Toledo, Spain), and was applied with south polarity to the motor cortex, over the hand area contralateral to the more affected side of the body (the center of the magnet was positioned 6.5 cm lateral to Cz). A non-magnetic metal cylinder, with the same size, weight and appearance of the magnet, was used for sham stimulation (MAG45s; Neurek SL, Toledo, Spain). tSMS or sham was applied for 10 minutes in each session. The patients’ blindness to experimental conditions was formally tested by asking them if they thought they had received the real intervention or the sham.

### Measurement of motor cortex excitability by TMS

Cortical excitability contralateral to the more affected side of the body was quantified by the amplitude of MEPs elicited by single-pulse TMS (duration 300μs) using a Magstim 200 magnetic stimulator (Magstim Co., Whitland, UK), which delivers monophasic single pulses. The D70mm Alpha figure-of-eight coil (Magstim Co., Whitland, UK) was held, with the handle pointing backwards and laterally at 45 deg from the midline, over the motor cortex at the optimum scalp position to elicit motor responses in the contralateral first dorsal interosseus (FDI). Intensities were expressed as a percentage of the maximum stimulator output. Surface electromyography (EMG) was recorded from the FDI by use of adhesive electrodes in a belly tendon montage. MEPs were amplified and filtered (bandwidth 3 Hz to 3 kHz) by D360 amplifiers (Digitimer, Welwyn Garden City, UK). Data were sampled at 10 kHz, collected on a computer and stored for later analysis using a CED 1401 A/D converter and Spike2 software (Cambridge Electronic Design, Cambridge, UK). Before each experimental session, the resting motor threshold (RMT) was assessed as the minimum stimulus intensity that produced a MEP with amplitude of at least 50 µV at rest. TMS intensity was then set at 120% RMT and remained constant throughout the session. At a rate of 10 stimuli/minute (0.17 Hz), 20 MEPs were recorded at baseline (2 min) and 100 MEPs were recorded after the intervention (10 min) (Fig. 1A). Note that RMT was separately assessed OFF and ON medication. Note that in our previous study in normal subjects, the effects of 10-min tSMS were significant only till 6 min post-stimulation^16^. Consequently, in the present study we only recorded MEPs for 10 min after the intervention.

### Data analysis

MEPs were quantified by peak-to-peak amplitude. Single-trial MEPs that exceeded 2 standard deviations from the session average or that were preceded by clear EMG activation were manually rejected (note that this process was performed blind to the real vs sham tSMS condition). The remaining single-trial MEPs were then averaged over 2-min time windows to obtain single-patient MEP amplitudes. Percent changes in MEP amplitudes were calculated by normalizing MEP amplitude to individual baseline values. The relative increment of tSMS plasticity after levodopa was calculated as (%changeON − %changeOFF)/%changeOFF, using the real sessions. With this measure, a dopamine-dependent increment of tSMS inhibitory plasticity (e.g. from −20% to −40%) would give a positive value (=1 in the example), whereas a dopamine-dependent paradoxical switch from inhibitory to excitatory plasticity (e.g. from −20% to +40%) would give a negative value (−3 in the example).

### Statistical analyses

The effects of the tSMS or sham on MEP amplitudes were assessed with three-way repeated-measures mixed ANOVA, with the following factors: MEDICATION (OFF, ON; between subjects), STIMULATION (real, sham; between subjects) and TIME (baseline, post 0–2 min, 2–4 min, 4–6 min, 6–8 min, 8–10 min), followed by follow-up ANOVAs and Dunnett post-hoc tests. Mean percent changes in MEP amplitudes were compared with student t-tests. The between-subjects variability of these changes was compared with two-sample F-test. Correlation between the relative increment of tSMS plasticity after levodopa and UPRS III score before levodopa administration was tested with Spearman correlation coefficient. All results were considered significant at p < 0.05.

## Results

None of the patients experienced any adverse event during or after the application of tSMS. Patients were not able to identify if the stimulation was real or sham. MEP amplitudes of an individual patient before and immediately after real and sham tSMS, OFF and ON medication, are provided in Fig. 1B. Overall, the application of tSMS for 10 min over the motor cortex significantly modulated the MEP amplitude in a dopamine-dependent way (three-way ANOVA, interaction real-sham x time x OFF-ON, F_5,215_ = 4.0, p = 0.0016; Fig. 1C).

In patients OFF medication, tSMS induced a significant modulation compared to sham (two-way follow-up ANOVA, interaction real-sham x time, F_5,110_ = 3.5, p = 0.0057). Specifically, MEP amplitude significantly decreased compared to baseline after tSMS (one-way follow-up ANOVA, time, F_5,50_ = 5.1, p = 0.0007) at 0–2 min (Dunnett, p = 0.0010), 2–4 min (p = 0.0010) and 4–6 min (p = 0.0229), whereas it did not change after sham (F_5,60_ = 1.3, p = 0.27). The average percent change of MEP amplitude at 0–6 min was −31.5 ± 26.2% after tSMS (n = 11) and +9.7 ± 21.8% after sham (n = 13; unpaired t-test: p = 0.0004). The average percent change of MEP amplitude at 0–6 min after tSMS was not significantly different compared to previously-published younger healthy subjects (Exp 2 in reference [16]; −21.4 ± 13.8%, n = 11; unpaired t-test: p = 0.27).

In patients ON medication, MEP amplitude significantly increased after the intervention (two-way follow-up ANOVA, time, F_5,105_ = 3.6, p = 0.0044), but possible differences between tSMS and sham did not reach significance (interaction real-sham x time, F_5,105_ = 1.7, p = 0.14). The average percent change of MEP amplitude at 0–6 min was +42.6 ± 66.6% after tSMS and +13.8 ± 21.8% after sham (unpaired t-test: p = 0.21).

The RMT did not change ON medication (36.6 ± 6.4) compared to OFF medication (37.7 ± 6.4, paired t-test: p = 0.28, n = 11). The between-patients variability of tSMS-induced changes was significantly greater ON medication compared to OFF medication (two-sample F-test, p = 0.0064; Fig. 1D). Interestingly, the relative increment of tSMS plasticity after levodopa ((ON-OFF)/OFF) was negatively correlated with the MDS-UPDRS III motor score before levodopa intake (n = 10; Spearman R = −0.69, p = 0.0282; Fig. 1E).

## Discussion

The main result of the present work is that the application of tSMS for 10 min over the motor cortex decreases cortical excitability – as measured by MEP amplitude – in patients with Parkinson’s disease after overnight withdrawal of dopaminergic therapy (OFF) but not after an acute dose of levodopa (ON). The between-patients variability of tSMS-induced changes was significantly greater ON medication. The variability ON medication could be partly explained by disease progression. These results suggest that tSMS induces dopamine-dependent changes of cortical excitability in patients with Parkinson’s disease.

### Methodological considerations

tSMS is a relatively new technique, thus not as consolidated as other NIBS techniques. The ability of tSMS to reduce MEP amplitude was initially demonstrated by us in healthy subjects^16^, and subsequently replicated by at least three independent groups^19, 21, 22^. tSMS has also been shown to modulate the excitability of the somatosensory cortex^20, 24^, and of the visual cortex in humans^18^, cats and monkeys^23^. As far as we know, this is the first tSMS study in patients with Parkinson’s disease. Our sample size is relatively small (13 patients). However, it offers a statistical power that is comparable to previous studies on dopamine-dependent changes of cortical plasticity in Parkinson’s disease^13, 15, 25, 26^. Similarly to previous works, we only tested one dose of levodopa and we did not use navigated TMS. The supposed advantage of navigated TMS vs non-navigated TMS remains controversial for the motor cortex^27, 28^. An important strength of our work, compared to most previous studies on cortical plasticity in Parkinson’s disease, is the inclusion of a sham condition in a randomized double-blind design. This excludes experimental biases, eliminates the possible confound of placebo-induced changes in cortical excitability^29^, and ensures that our results are robust to possible between-sessions and between-patients variability in TMS coil and/or tSMS positioning. It should be noted that tSMS-ON sessions always followed a sham-OFF session, with at least one-hour separation between the two. The sham-OFF session did not induce any significant changes on cortical excitability, so the possibility of carryover effects of the sham-OFF session onto the tSMS-ON session seems unlikely. Future studies should establish the impact of tSMS duration on the strength and duration of tSMS-induced plasticity OFF and ON medication, and the dose-dependence of levodopa effects, in larger cohorts of patients.

### tSMS plasticity OFF medication

The ability of tSMS to reduce cortical excitability in patients OFF medication is similar to previous observations in younger healthy subjects^16, 19, 21, 22^, and is consistent with the recent results obtained with continuous theta-burst stimulation (cTBS) in patients with Parkinson’s disease^15^. LTD-like plasticity induced by cTBS in patients OFF medication is normal in non-fluctuating patients, is somewhat impaired with the appearance of motor fluctuations and is lost only when dyskinesias are observed after the morning dose of drugs^15^. In our patients, overt dyskinesias were not observed after 150–200% of the morning dose during the experimental sessions. Our results are thus in line with the cTBS results obtained in patients with little or mild motor complications^15^. At first glance, this preserved LTD-like plasticity might seem to contrast with the loss of long-term depression (LTD) observed at both striatal and cortical levels in animal models of dopamine depletion^30, 31^. But the contrast is solved by the observation that cTBS is not able to reduce cortical excitability in *de novo* untreated patients – and a single dose of levopopa is not able to rescue this ability^14^. The most likely interpretation of these findings, already proposed by Kishore *et al*.^15^, is thus that dopamine depletion disrupts cortical plasticity induced by inhibitory NIBS techniques, but the long-duration response to levodopa^32^ – which is still present after overnight withdrawal of dopaminergic drugs – is able to rescue it, leading to the ‘normal’ reduction of cortical excitability with tSMS in our patients OFF medication.

### tSMS plasticity ON medication

After an acute high dose of levodopa (200–250 mg), tSMS lost its ability to reduce cortical excitability, again in line with previous findings obtained with cTBS^15^. The absence of RMT changes ON vs OFF medication, consistent with previous studies^15, 26, 33^, excludes possible gross alterations of cortical excitability due to the acute administration of levodopa. Previous experiments in healthy subjects showed that a high dose of levodopa (200 mg) blocks inhibitory cortical plasticity induced by cathodal transcranial direct current stimulation (tDCS)^34^. The loss of inhibitory cortical plasticity after levodopa intake in patients with Parkinson’s disease might thus be a normal physiological response. However, we also found that the relative increment of tSMS plasticity induced by levodopa was negatively correlated with the UPDRS III score before levodopa intake, i.e. the more advanced the patient, the more likely it was to observe a switch from inhibitory tSMS plasticity OFF medication to paradoxical facilitatory plasticity ON medication. The higher variability of tSMS plasticity ON medication could thus be at least partly explained by the progression of the disease, in agreement with the cTBS findings^15^. Interestingly, disease progression enhances the changes in synaptic dopamine levels induced by the same dose of levodopa^35^, particularly in patients that will develop motor complications^36^. Excessive levels of synaptic dopamine and paradoxical facilitatory plasticity in response to inhibitory NIBS techniques ON medication might thus represent two sides of the same pathophysiological progression of the disease^15^.

### Pathophysiological and practical implications

Overall, the present tSMS results are consistent with previous cTBS results in patients with Parkinson’s disease^15^. This similarity is important because of the marked biophysical differences between the two techniques. cTBS uses a large rapidly-changing magnetic field that induces relatively strong electric currents in the brain. These currents might activate corticostriatal neurons^37, 38^, thereby leaving open the possibility of the dopamine-dependent cortical plasticity induced by cTBS to be a non-specific epiphenomenon of the dopaminergic control of striatal plasticity. Conversely, tSMS uses moderate-intensity static magnetic fields that are believed to alter the function of membrane ion channels due to the diamagnetic anisotropic properties of membrane phospholipids^39–41^. Even though induction of electric currents with tSMS cannot be completely ruled out due to brain pulsation, strong activation of corticostriatal neurons seems unlikely. The similar dopamine-dependent plasticity obtained with the two techniques might thus be genuinely cortical. This view is in line with recent experiments in animal models of dopamine depletion showing direct dopaminergic regulation of motor cortex plasticity^31^.

On a more practical level, tSMS is more portable, easy-to-apply and inexpensive compared to cTBS (and to low-frequency rTMS). The ability of tSMS to induce dopamine-dependent changes in cortical excitability makes this technique attractive in Parkinson’s disease not only for pathophysiological studies, but also for possible treatments. Admittedly, our study was not designed to investigate clinical effects induced by tSMS, but these effects – if any – are unlikely to be relevant with the short-lasting neurophysiological results reported here. In fact, the effects of 10-min tSMS were already wearing off after 6 min in our patients OFF medication, similarly to what we observed in healthy subjects. Future studies are needed in the development of longer-lasting tSMS protocols to allow this NIBS technique to be translated from pathophysiological studies toward clinical applications.

### Conclusions

In conclusion, our results suggest a dopamine dependency of cortical changes induced by tSMS in Parkinson’s disease and encourage the application of this promising NIBS technique – inexpensive, safe, portable and DBS-compatible – for pathophysiological research in movement disorders.
